# Comparison of procedures for RNA-extraction from peripheral blood mononuclear cells

**DOI:** 10.1371/journal.pone.0229423

**Published:** 2020-02-21

**Authors:** Antonio Rodríguez, Hans Duyvejonck, Jonas D. Van Belleghem, Tessa Gryp, Leen Van Simaey, Stefan Vermeulen, Els Van Mechelen, Mario Vaneechoutte

**Affiliations:** 1 Laboratory Bacteriology Research, Department of Diagnostic Sciences, Faculty of Medicine and Health Sciences, Ghent University, Ghent, Belgium; 2 Department of Biosciences, Faculty of Education, Health and Social Work, University College Ghent, Ghent, Belgium; 3 Division of Infectious Diseases and Geographic Medicine, Department of Medicine, Stanford University School of Medicine, Stanford, California, United States of America; The Ohio State University, UNITED STATES

## Abstract

RNA quality and quantity are important factors for ensuring the accuracy of gene expression analysis and other RNA-based downstream applications. Thus far, only a limited number of methodological studies have compared sample storage and RNA extraction procedures for human cells. We compared three commercially available RNA extraction kits, *i*.*e*., (NucliSENS) easyMAG, RNeasy (Mini Kit) and RiboPure (RNA Purification Kit–blood). In addition, additional conditions, such as storage medium and storage temperature of human peripheral blood mononuclear cells were evaluated, i.e., 4 °C for RNAlater or -80 °C for QIAzol and for the respective cognate lysis buffers; easyMAG, RNeasy or RiboPure. RNA was extracted from aliquots that had been stored for one day (Run 1) or 83 days (Run 2). After DNase treatment, quantity and quality of RNA were assessed by means of a NanoDrop spectrophotometer, 2100 Bioanalyzer and RT-qPCR for the *ACTB* reference gene. We observed that high-quality RNA can be obtained using RNeasy and RiboPure, regardless of the storage medium, whereas samples stored in RNAlater resulted in the least amount of RNA extracted. In addition, RiboPure combined with storage of samples in its cognate lysis buffer yielded twice as much RNA as all other procedures. These results were supported by RT-qPCR and by the reproducibility observed for two independent extraction runs.

## Introduction

RNA expression level is a good indicator of the physiological status of cells and reveals the cell response under different stress conditions such as those encountered during host-pathogen interactions. High quality and quantity of extracted RNA is crucial for downstream applications such as reliable gene expression quantification through quantitative PCR [1] and RNA-sequencing (RNA-seq) [2,3]. Unfortunately, proper RNA quality controls including RNA integrity are lacking in many studies [4,5,6,7,8].

Prior to RNA extraction, the appropriate storage of samples is of utmost importance as well. First, RNA is prone to degradation and since different RNAs can have different stability, this may influence the gene expression pattern [9]. On the other hand, transcription and translation can continue to some extent after collection of the samples, and therefore the final RNA composition may not reliably represent the relative RNA content as present at the moment of collection. Collected samples are usually stored cryogenically by submerging the samples in liquid nitrogen (- 180 °C). However, cryopreservation in a clinical environment is not always possible or practical. Different methods have therefore been developed to avoid both RNA degradation and RNA transcription. For example, several storage media that are commercially available have been used for blood samples, such as PAXgene Blood RNA tubes (PreAnalytiX Qiagen/BD, Hombrechtikon, Switzerland), Tempus Blood RNA tubes (Applied Biosystems, Foster City, CA) and RNAlater Stabilization Reagent (Thermo Fisher Scientific, Waltham, MA) [10,11,12,13,14,15,16]. In this study, we used RNAlater because it is a common stabilization reagent for RNA in different cells and tissues, whereas the former two are more commonly used for blood and their performance in other cell type or tissues is uncertain.

Obtaining high-quality RNA also depends on the RNA extraction kit. Some kits are based on phenol-chloroform extraction, such as the RiboPure RNA Purification Kit—blood (Thermo Fisher Scientific) and TRI Reagent (Sigma-Aldrich, San Luis, MO), which contain guanidine thiocyanate to denature cellular components and to inhibit RNase activity. Other kits are based on silica spin columns, such as the NucleoSpin RNA Blood Kit (Macherey-Nagel, Düren, Germany) and the RNeasy Mini Kit (Qiagen, Hilden, Germany), which also use guanidine thiocyanate and β-mercaptoethanol, respectively, to inactivate RNases. Finally, other extractions are based on magnetic silica particles that capture nucleic acids, *e*.*g*. the NucliSENS easyMAG platform (bioMérieux, Marcy-l'Étoile, France).

Regardless of the storage condition and RNA extraction procedure used, it is necessary to determine not only the quality but also the quantity of RNA prior to downstream analysis. Although it has been shown that 500 picograms is enough for cDNA synthesis [17], it is recommended to start from at least 100 nanograms for other downstream applications such as RNA-seq [18].

Studies comparing different storage conditions and RNA extraction procedures that comprehensively analyze both RNA quality and quantity are therefore needed. In addition, results need to be somehow quantified and directly measured in order to facilitate comparison with other studies. For example, one study previously analyzed RNA yield after different storage conditions, and quantified RNA yield indirectly with Cq values obtained during reverse transcription and amplification (RT-qPCR) [19], such that proper comparison of results obtained in other studies is only possible when the same primers and PCR conditions are used. The use of methods that allow direct quantification of RNA yield and quality, such as bioanalyzers, may facilitate comparison between RNA extraction procedures from different studies.

In this study, we compared the effect of storage of human peripheral blood mononuclear cells (PBMCs) in different storage media during two different time periods (one day vs. 83 days). We also compared three different RNA extraction kits. We analyzed RNA quality (as RNA purity and RNA integrity) and RNA yield using a NanoDrop spectrophotometer ND-1000 (Isogen Life Science, Utrecht, The Netherlands), a 2100 Bioanalyzer, and through RT-qPCR of the *ACTB* gene. Finally, RNA quality and yield were compared to determine which combination procedure of storage and extraction kit provided the best results.

## Materials and methods

All methods were carried out in accordance to relevant guidelines and regulations and all experimental protocols were approved by the ethical committee of the University of Ghent (EC/2016/0192).

Raw data containing all measurements, averages and standards deviations of the main figures and tables used in this study are shown in S1 File.

### General set up of the comparison

**Fig 1:**
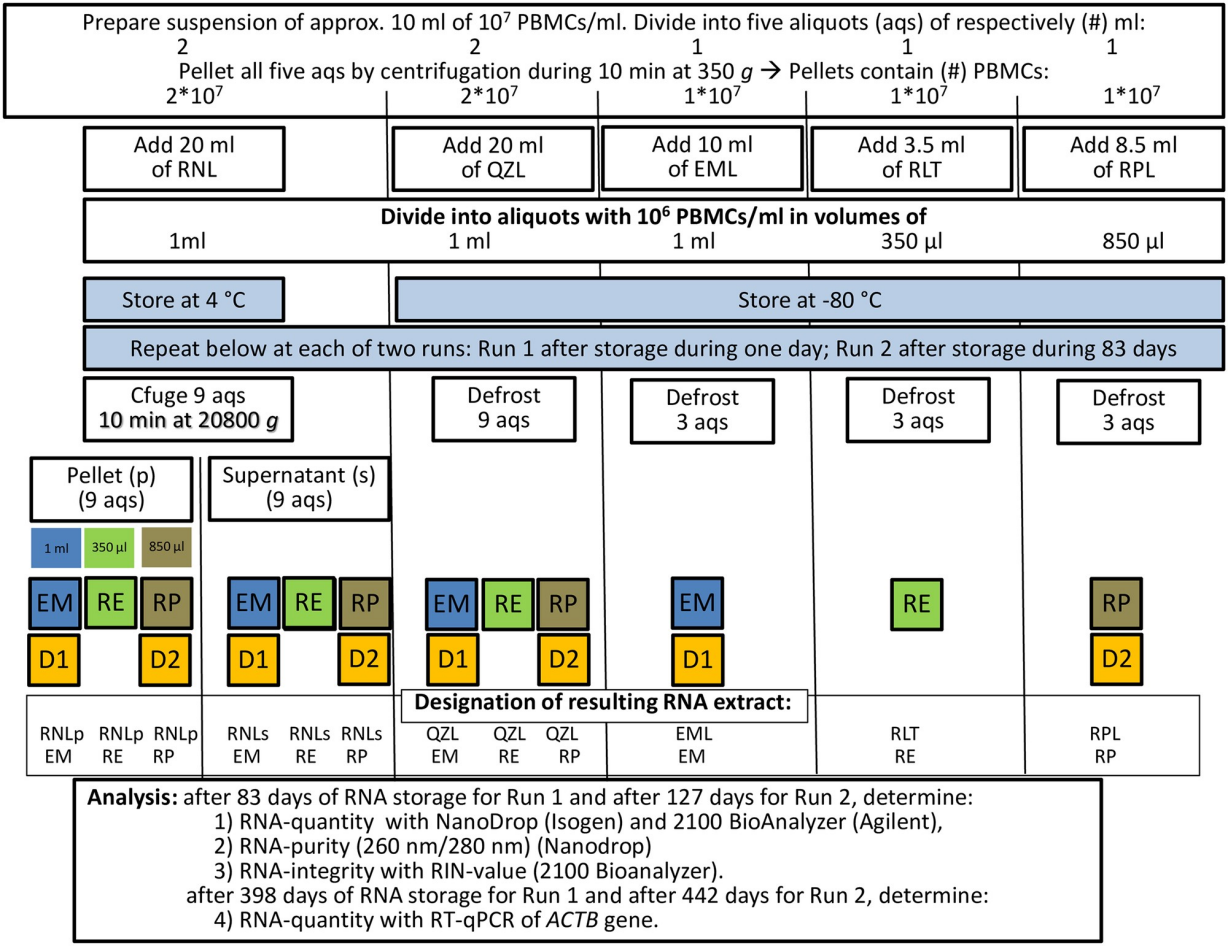
Study set up. Preparation of 10^6^ PBMC aliquots, storage, RNA extraction and analysis of RNA quality, integrity and quantity. Storage medium: EML: NucliSENS easyMAG lysis buffer; QZL: QIAzol; RLT: RNeasy Mini Kit lysis buffer; RNLp: RNAlater pellet; RNLs: RNAlater supernatant; RPL: RiboPure RNA Purification Kit–blood lysis buffer. RNA extraction kits: EM: NucliSENS easyMAG; RE: RNeasy Mini Kit; RP: RiboPure RNA Purification Kit–blood. DNase treatment: D1, D2: as described in the M&M section. For the RE RNA Kit, DNase treatment was included during the extraction procedure, as described in the M&M section.

The setup of this study is illustrated in Fig 1 and S1 Table. To assess the quantity and the quality of RNA that could be extracted from PBMCs, we stored aliquots containing 10^6^ PBMCs for two different time periods (1 day and 83 days) in five different storage media, *i*.*e*., (1) at 4 °C in RNAlater (RNL) or at -80 °C in (2) QIAzol Lysis Reagent (QZL, Qiagen), or in the cognate lysis buffers, *i*.*e*., (3) easyMAG lysis buffer (EML) or (4) RNeasy Mini Kit lysis buffer (RLT) or (5) RiboPure RNA Purification Kit—blood, lysis buffer (RPL). In addition, three commercial RNA extraction kits were compared, *i*.*e*., NucliSENS easyMAG (EM), RNeasy Mini Kit (RE) and RiboPure RNA Purification Kit—blood (RP).

Since RNAlater (RNL) is not a lysis buffer, it must be removed to avoid interference with the cognate lysis buffer and downstream procedures, such as RNA extraction. Therefore, after storage in RNL, the aliquots were centrifuged and RNA was extracted from the pellet (RNLp). RNA was also extracted from the supernatant (RNLs) to evaluate crossover of RNA to the supernatant during centrifugation of the aliquots, which would lead to underestimation of the RNA quantity.

This resulted in a total of 12 procedures (4 sample storage conditions in combination with 3 RNA extraction kits), as indicated in Fig 1 and S1 Table. These procedures were named by combining the abbreviation of the storage condition (RNLp, RNLs, QZL, and EML or RLT or RPL) with that of the commercial RNA extraction kit (EM, RE, RP).

All 12 procedures were carried out for triplicate aliquots, and after storage for one day (Run 1) and for 83 days (Run 2).

RNA quantity and quality were assessed, after DNase treatment of the RNA extracts, using a NanoDrop ND-1000 and a 2100 Bioanalyzer for each RNA extract, after storage of the RNA for 83 days (Run 1) and for 127 days (Run 2), and by means of RT-qPCR for the *ACTB* (reference) gene after storage of the RNA for 398 days (Run 1) and 442 days (Run 2), respectively.

### Preparation of peripheral blood mononuclear cells

PBMCs were isolated from a buffy coat derived from one donor from Rode Kruis Vlaanderen (Ghent, Belgium), as previously described in [20].

Cells were centrifuged again at 350 *g* for 10 min, the supernatant was removed, and cells were resuspended in HBSS to reach a final concentration of 10^7^ PBMCs/ml.

### Standardized preparation of aliquots at 10^6^ cells/ml in five different storage media

The PBMCs were stored in five different storage media, whereby each of the five batches was divided into different aliquots prior to storage, in order to minimize the number of freeze-thaw cycles. Prior to storage, the suspension of 10^7^ PBMCs/ml was divided in five parts (Fig 1, S1 Table). These five aliquots were centrifuged at 350 *g* for 15 min and the pellets were resuspended in one of the five different storage media (RNL, QZL, EML, RLT and RPL), at 10^6^ PBMC/ml. The five batches were aliquoted in numbers and volumes that were appropriate for each protocol. All aliquots were stored at -80 °C, or at 4 °C for RNL, until the three RNA extraction procedures were carried out.

### RNA extraction with three different kits

The three RNA extraction kits (EM, RE, RP) were each carried out on two separate dates, *i*.*e*., after one day (Run 1) and after 83 days (Run 2) of storage. Each RNA extraction kit was carried out in triplicate, *i*.*e*., starting from three separately stored aliquots per storage condition.

For each run, on the day of the RNA extractions, the RNL-stored aliquots were centrifuged for 10 min at 20,800 *g* to remove RNL and then the pellet of cells (RNLp) was resuspended in the cognate lysis buffer of each kit. Removal of RNL is needed to avoid dilution of and interference with the cognate lysis buffers, in order to maximize cell lysis. In addition, we also extracted RNA from the RNLs to assess to what extent RNA was lost into the supernatant (RNLs)–which is usually discarded.

For the EM RNA extraction kit, the manufacturer advises to start the extraction on the apparatus in 2-ml volumes. To achieve this volume for the RNLp, 1 ml of EML was added to the pellet. This was not needed for RNLs or for cells that had been stored in QZL or in the cognate lysis buffer (EML), which were already in a 1 ml volume. Subsequently, EM RNA extraction was started by adding 1 ml of EML to each of 12 empty cartridges. Thereafter, the 12 one-ml aliquots, *i*.*e*., four series (RNLp, RNLs, QZL and EML) of three replicates each, were added to the cartridges, to reach the required 2-ml starting volume. After incubation for 10 min at room temperature, 100 μl of silica v/v EM Extraction Buffer 3 was added to each cartridge and processed on the easyMAG. The RNA was eluted in 35 μl of EM elution buffer.

For the RE RNA extraction kit, the cells in the RNLp were disrupted by mixing with 350 μl of RLT, that had been supplemented with 143 mM β-mercaptoethanol. The disrupted RNLp, as well as the RNLs, QZL and RLT aliquots, were further homogenized by pipetting directly onto a QIAshredder spin column, that was placed in a 2 ml collection tube, and by subsequent centrifugation for 2 min at full speed. All aliquots were mixed with 350 μl of 70% ethanol for RNLp and RLT, and with 1 ml of 70% ethanol for RNLs and QZL to the homogenized lysate, in order to keep a 1:1 ethanol/lysate ratio.

For the RP RNA extraction kit, 300 μl of acid-Phenol:Chloroform was added to all 12 samples, with PBMCs in 1 ml of QZL, in 1 ml of RNLs or in 850 μl of RPL, respectively. Pellets from RNLp were disrupted by adding 850 μl of RPL and vortexed vigorously to resuspend and lyse the cells.

### DNase digestion of the RNA extracts

Contaminating DNA was removed from each RNA extract by means of DNase treatment, according to the manufacturer’s recommendations.

DNase digestion of the EM RNA extracts (35 μl) (Fig 1D1) was carried out by adding 7 μl of RQ1 RNase-Free DNase 10X reaction buffer, 7 μl of RQ1 RNase-free DNase (1 U/μg RNA) (Promega Benelux, Leiden, the Netherlands) and 21 μl of nuclease-free water (total volume of 70 μl) and incubation for 30 min at 37 °C. The reaction was stopped by adding 7 μl of RQ1 DNase Stop Solution and incubation for 10 min at 65 °C (Fig 1D1). The final volume after DNase treatment and inactivation was therefore 77 μl.

The RE RNA extraction procedure comprises an on-column DNase treatment using RNase-free DNase (Qiagen), as described by the manufacturer.

DNase digestion of the RP RNA extracts (Fig 1D2) was carried out by adding 5.5 μl of 20x DNase buffer and 1 μl of 8 U DNase I/μl (RiboPure) to the 100 μl of RNA eluates, mixing gently but thoroughly and incubation for 30 min at 37 °C. The DNase reaction was inactivated by adding 20 μl of DNase inactivation reagent and incubation for 2 min at room temperature. The DNase inactivation reagent was pelleted by centrifugation for 1 min at 20,800 *g*, and the supernatant was transferred to a new RNase-free tube. The final volume after DNase treatment and inactivation was 126.5 μl.

### Assessment of RNA purity and RNA integrity of the RNA extracts

RNA purity was assessed by determination of the ratio for absorbance at 260 nm vs. absorbance at 280 nm (A_260 nm_/A_280 nm_) using a NanoDrop.

RNA integrity was also assessed with the 2100 Bioanalyzer determining RIN-values by gel electrophoresis.

### Assessment of RNA quantity of the RNA extracts

The RNA yield was assessed by means of NanoDrop and the 2100 Bioanalyzer in combination with the RNA 6000 Nano kit (Agilent) and by means of RT-qPCR for the *ACTB* gene.

For RT-qPCR, cDNA was prepared by adding 5 μl of RNA extract to a final volume of 20 μl of the RevertAid RT Kit (Thermo Fisher Scientific) according to the manufacturer’s instructions. Two μl of the cDNA was subsequently added to a total reaction volume of 10 μl PCR mix, consisting of 0.5 μM of each β-actin primer [21], 2 mM of MgCl_2_ and LightCycler 480 High Resolution Melting Master Mix (Roche Applied Sciences, Indianapolis, IN, USA). Amplification was carried out on a LightCycler 480 (Roche) using the following program: pre-incubation for 30 s at 95 °C and amplification for 45 cycles of 30 s at 95 °C, 10 s at 56 °C and 30 s at 72 °C, after which a high resolution melting curve was generated, using the following protocol: 5 s at 95 °C, 1 min at 58 °C, followed by a gradual increase in temperature from 60 °C to 97 °C, using a ramp rate of 0.02 °C per s. Results were analyzed with the standard LightCycler 480 Software, version 1.5 (Roche).

### Comparison of different combinations of storage in EML or RPL and RNA extraction with EM or RP

We aimed to assess which part of the RNA extraction procedure *i*.*e*., the storage medium or the RNA extraction kit, was most important for the procedure that resulted in the highest RNA yield, *i*.*e*., the RPL-RP procedure. Therefore, we performed the EM RNA extraction kit after storage of the PBMCs in 2 ml of RPL instead of 2 ml of EML, and the RP RNA extraction kit after storage of the PBMCs in 850 μl of EML instead of 850 μl of RPL.

### Statistical analysis

For statistical comparisons of sample storage conditions, RNA extraction kits, and of instruments to analyze RNA (NanoDrop versus 2100 Bioanalyzer), three independent extractions were considered and data were analyzed using the linear mixed model for repeated measures, followed by Bonferroni’s multiple testing correction. The *t* test for paired samples was applied to compare results from both runs, using the IBM SPSS Statistics software v 25.0 (IBM, Armonk, NY, USA).

## Results

### Comparison of purity and integrity of RNA, obtained with different RNA extraction procedures

All three extraction kits (EM, RE and RP) yielded RNA with A_260 nm_/A_280 nm_ ratios close to 2.0 (Table 1), indicative of pure RNA [22], except for the lower value of 1.6 for the RNL-RE procedure in Run 1.

**Table 1:** RNA purity (NanoDrop) and RNA integrity (2100 Bioanalyzer) for three different RNA extraction kits after storage of PBMCs in three different storage media^a^.

PBMC storage medium	RNA extraction kit	NanoDrop A_260_/A_280_^b^		2100 Bioanalyzer RIN^c^	
		Run 1 (day 1)	Run 2 (day 83)	Run 1 (day 1)	Run 2 (day 83)
QIAzol	NucliSENS easyMAG	2.0 ± 0.2	2.0 ± 0.1	1.0 ± 0.0	1.0 ± 0.0
RNAlater		2.0 ± 0.2	2.3 ± 0.2	1.0 ± 0.0	1.0 ± 0.0
easyMAG lysis buffer (EML)		2.1 ± 0.3	2.1 ± 0.2	1.0 ± 0.0	1.0 ± 0.0
QIAzol	RNeasy Mini Kit	1.9 ± 0.1	1.9 ± 0.1	9.4 ± 0.8	9.2 ± 0.3
RNAlater		1.6 ± 0.2	2.2 ± 0.3	8.2 ± 0.6	7.9 ± 0.6
RNeasy Mini Kit lysis buffer (RLT)		1.9 ± 0.2	2.1 ± 0.1	8.7 ± 0.5	8.8 ± 0.5
QIAzol	RiboPure Kit—Blood	2.0 ± 0.1	2.1 ± 0.2	1.0 ± 0.0	7.9 ± 0.5
RNAlater		2.1 ± 0.1	2.1 ± 0.0	8.1 ± 0.5	8.4 ± 0.6
RiboPure Kit–Blood lysis buffer (RPL)		2.0 ± 0.0	2.0 ± 0.1	8.9 ± 0.6	8.6 ± 0.3

^a^: Results are means and standard deviations of three independent extractions.

^b^: A value of ~2.0 is generally accepted as indicating that RNA is free of proteins.

^c^: The RIN value is reported on a scale of 1 to 10, whereby values above 7 are considered to represent high quality and non-degraded RNA.

Using the 2100 Bioanalyzer, RIN values higher than 7 were observed for the RE and RP extraction kits, except for QZL-RP in Run 1 (Table 1), which is probably due to a technical error, because the value for this procedure during Run 2 was 7.87 ± 0.49. However, all EM-related procedures yielded RIN values of 1. More detailed electropherograms and gel images of Run 1 and Run 2 are shown in S1 and S2 Figs, respectively.

### Assessment of RNA quantity with NanoDrop vs. 2100 Bioanalyzer

**Fig 2:**
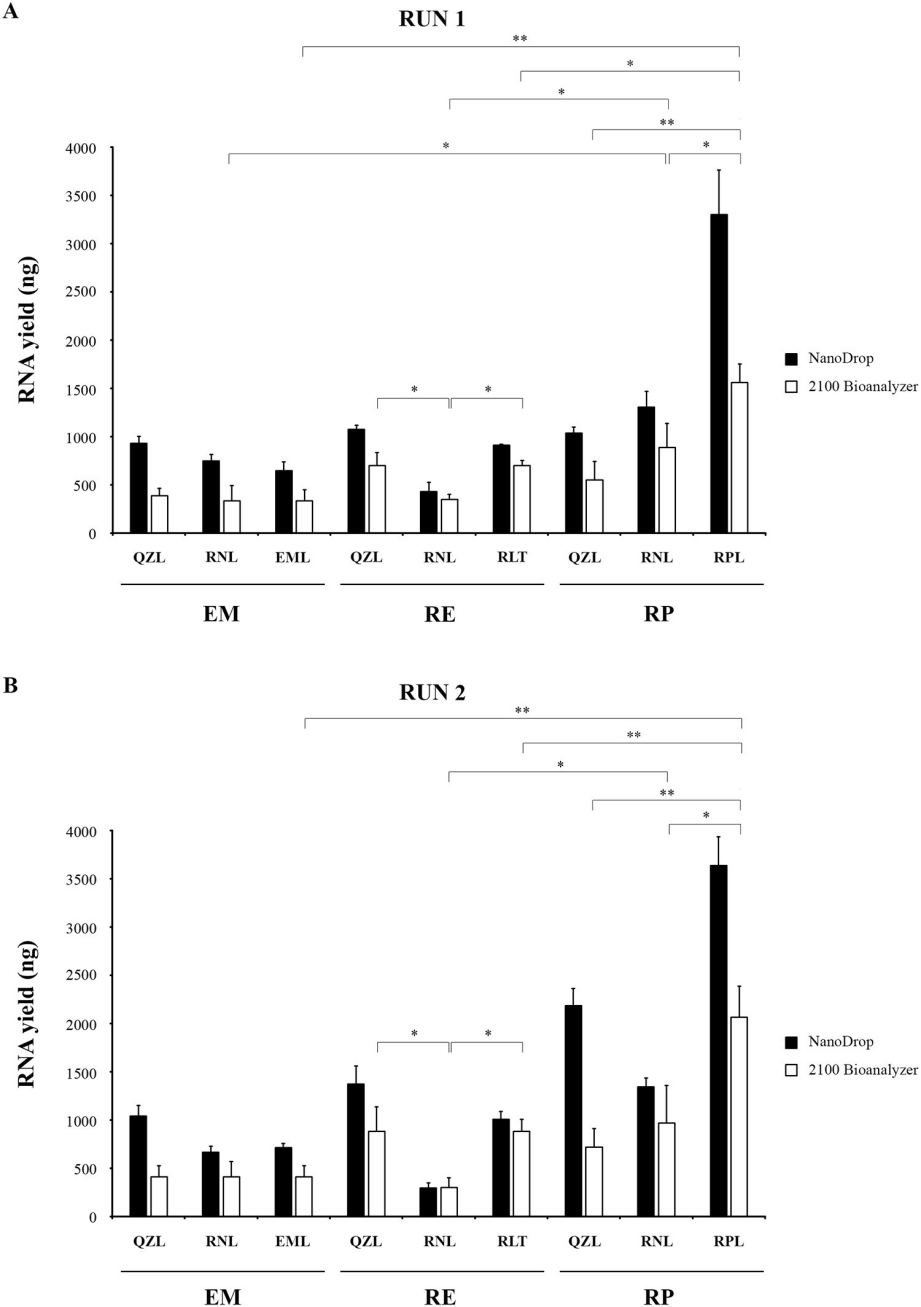
RNA yield according to NanoDrop spectrophotometer (black bars) and 2100 Bioanalyzer (white bars) for three different extraction kits after storage of PBMCs in three different storage media. A. Run 1. Storage of PBMCs for one day. RNA quantification after storage of RNA for 83 days. B. Run 2. Storage of PBMCs for 83 days. RNA quantification after storage of RNA for 127 days. Error bars represent the standard deviations of results from three independent extractions. Statistically significant differences are marked with one (*p* < 0.05) or two (*p* < 0.005) asterisks (Linear mixed model for repeated measures). EM: NucliSENS easyMAG; RE: RNeasy Mini Kit; RP: RiboPure RNA Purification Kit–blood; QZL: QIAzol; RNL: RNAlater; EML: easyMAG lysis buffer; RLT: RNeasy lysis buffer; RNL: RNAlater; RPL: RiboPure RNA Purification Kit–blood lysis buffer.

The RNA measurements with Nanodrop were 1.1- to 2.5-fold higher (*p* < 0.05) than those obtained with the 2100 Bioanalyzer (Fig 2, Table 2). Despite these absolute differences, we observed a strong congruence. For example, RPL-RP clearly yielded most RNA, according to both NanoDrop and 2100 Bioanalyzer, compared to all other extraction procedures and storage conditions (Fig 2).

**Table 2:** RNA yield of different RNA extraction procedures according to NanoDrop and 2100 Bioanalyzer.

RNA extraction procedure	NanoDrop (ND)		2100 Bioanalyzer (BA)		ND vs. BA	
	RNA yield (ng)		RNA yield (ng)		*p* value	
	Run 1	Run 2	Run 1	Run 2	Run 1	Run 2
QZL-EM	929.1 ± 72.2	1038.5 ± 111.7	385.0 ± 77.0	410.7 ± 117.6	0.020	0.037
RNL-EM	745.6 ± 69.1	667.3 ± 58.8	333.7 ± 160.3	410.7 ± 160.3	0.035	0.057
EML-EM	647.6 ± 89.9	714.3 ± 40.9	333.7 ± 117.6	410.7 ± 117.6	0.030	0.044
QZL-RE	1076.7 ± 42.3	1371.0 ± 186.5	700.0 ± 132.3	883.3 ± 251.7	0.020	0.015
RNL-RE	432.8 ± 92.0	296.7 ± 51.3	350.0 ± 50.0	300.0 ± 100.0	0.418	0.921
RLT-RE	913.3 ± 9.0	1006.2 ± 84.9	700.0 ± 50.0	883.3 ± 125.8	0.017	0.419
QZL-RP	1037.3 ± 61.4	2185.9 ± 178.5	548.2 ± 193.2	716.8 ± 193.2	0.072	< 0.001
RNL-RP	1307.2 ± 160.7	1343.4 ± 90.7	885.5 ± 253.0	969.8 ± 386.5	0.036	0.171
RPL-RP	3299.5 ± 459.1	3634.8 ± 301.0	1560.2 ± 193.2	2066.2 ± 318.4	0.029	0.019

Results are means and standards deviation (in ng) from three independent extractions.

*p* values were calculated using the *t* test for paired samples.

Run 1. Storage of PBMCs for one day. RNA quantification after storage of RNA for 83 days

Run 2. Storage of PBMCs for 83 days. RNA quantification after storage of RNA for 127 days

Given the strong congruence between both analysis methods and taking into consideration that the higher values reported by NanoDrop probably resulted from the fact that this method also measured (oligo)nucleotides, further comparisons were made between procedures and runs only on the basis of 2100 Bioanalyzer results.

### Comparison of RNA yield between RNA extraction runs

**Fig 3:**
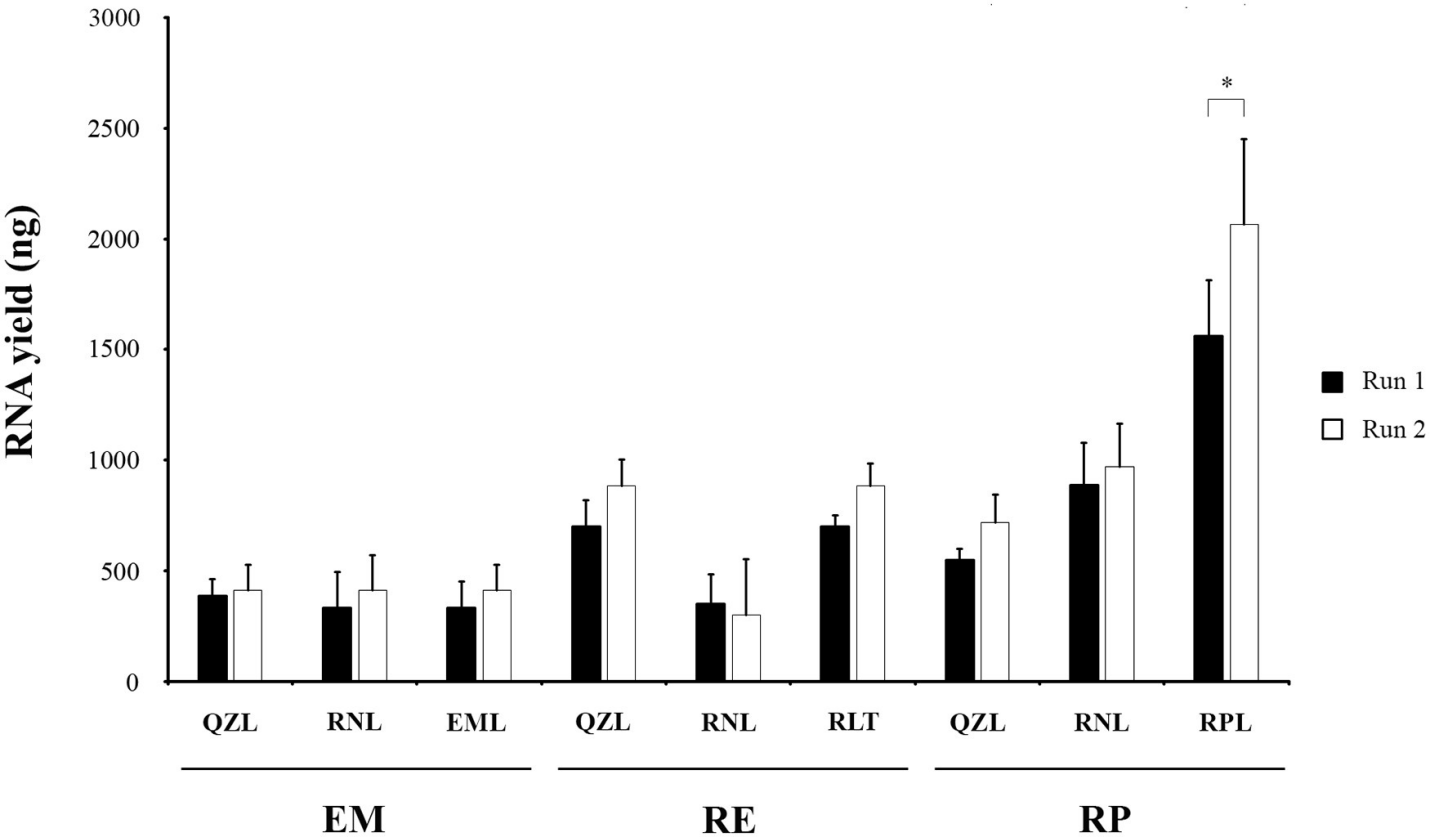
RNA yield according to 2100 Bioanalyzer from PBMCs stored in three different storage media and after three different RNA extraction kits performed on two separate dates (Run 1 and Run 2). Error bars represent the standard deviations of results from three independent extractions. Statistically significant differences are marked with an asterisk (*p* < 0.05) (*t* test for paired samples). EM: NucliSENS easyMAG; RE: RNeasy Mini Kit; RP: RiboPure RNA Purification Kit–blood; QZL: QIAzol; RNL: RNAlater; EML: easyMAG lysis buffer; RLT: RNeasy lysis buffer; RNL: RNAlater; RPL: RiboPure RNA Purification Kit–blood lysis buffer.

When comparing two runs of RNA extraction, carried out after different periods of storage of the PBMCs, *i*.*e*. after one day and after 83 days, no significant differences were observed with regard to yield, except for the RPL-RP procedure, which however yielded most RNA in both runs (Fig 3, Table 3).

**Table 3:** RNA quantification according to NanoDrop spectrophotometer, 2100 Bioanalyzer and RT-qPCR for the *ACTB* gene, for two runs of three different RNA extraction kits after storage of PBMCs in three different storage media.

PBMC Storage medium	RNA extraction kit (elution volume; final volume (μl))	NanoDrop		2100 Bioanalyzer		RT-qPCR	
		RNA concentration (ng/μl)*		RNA concentration (ng/μl)*		Cq values^●^	
		Run 1	Run 2	Run 1	Run 2	Run 1	Run 2
QIAzol	easyMAG (35; 77)	12.1 ± 0.9	13.5 ± 1.5	5.0 ± 1.0	5.3 ± 1.5	29.1 ± 0.1	35.3 ± 0.5
RNAlater p		9.7 ± 0.9	8.7 ± 0.8	4.3 ± 2.1	5.3 ± 2.1	30.8 ± 1.0	33.7 ± 1.1
RNAlater s		5.6 ± 0.9	6.3 ± 0.9	3.3 ± 1.5	7.3 ± 0.6	27.3 ± 0.2	29.7 ± 1.8
EML		8.4 ± 1.2	9.3 ± 0.5	4.3 ± 1.5	5.3 ± 1.5	29.4 ± 0.4	31.4 ± 0.8
QIAzol	RNeasy (50; 50)	21.5 ± 0.8	27.4 ± 3.7	14.0 ± 2.6	17.7 ± 5.0	27.7 ± 0.1	27.5 ± 0.5
RNAlater p		8.7 ± 1.8	5.9 ± 1.0	7.0 ± 1.0	6.0 ± 2.0	29.7 ± 0.5	31.1 ± 0.5
RNAlater s		3.6 ± 1.3	3.1 ± 0.3	5.0 ± 1.7	3.7 ± 1.2	> 40	> 40
RLT		18.3 ± 0.2	20.1 ± 1.7	14.0 ± 1.0	17.7 ± 2.5	27.5 ± 0.2	26.8 ± 0.3
QIAzol	RiboPure (100; 126.5)	8.2 ± 0.5	17.3 ± 1.4	4.3 ± 1.5	5.7 ± 1.5	36.7 ± 0.1	31.0 ± 0.4
RNAlater p		10.3 ± 1.3	10.6 ± 0.7	7.0 ± 2.0	7.7 ± 3.1	31.4 ± 0.2	31.6 ± 0.4
RNAlater s		2.3 ± 0.4	2.2 ± 0.5	2.3 ± 0.6	2.0 ± 1.0	> 40	> 40
RPL		26.1 ± 3.6	28.7 ± 2.4	12.3 ± 1.5	16.3 ± 2.5	28.9 ± 0.6	29.4 ± 0.3

*Run 1. Storage of PBMCs for one day. RNA quantification after storage of RNA for 83 days

Run 2. Storage of PBMCs for 83 days. RNA quantification after storage of RNA for 127 days

^●^Run 1. Storage of PBMCs for one day. RT-qPCR was performed after storage of RNA for 398 days

Run 2. Storage of PBMCs for 83 days. RT-qPCR was performed after storage of RNA for 442 days

### Comparison of RNA yield after storage of PBMCs in RNA later, QIAzol and cognate lysis buffers

We next analyzed which storage medium provided the highest RNA yield for each of the RNA extraction kits considered (Fig 2). For EM, no significant differences were observed after storage in RNL, QZL or the cognate EML. For RE, lowest yields were obtained after storage in RNL, while no statistical differences were observed between QZL and RLT. Finally, for RP, RPL storage provided a significantly higher yield than storage in RNL or QZL (*p* < 0.05).

### Comparison of RNA yield among RNA extraction procedures

Because, with regard to RNA yield, the best storage medium (RNL, QZL or cognate lysis buffer (EML, RTL, or RPL)) also depended upon the RNA extraction kit, we enquired which RNA extraction procedure resulted in the highest RNA yield. For this purpose, the combinations of each of the storage media with each of the RNA extraction kits was compared (Fig 2). For storage in QZL, RE resulted in a higher RNA yield than EM (*p* < 0.05), while no statistical differences were observed between RE and RP, nor between EM and RP. For both, storage in RNL and storage in cognate buffer, RP yielded most RNA (*p* < 0.05), when compared to EM and RE, which were similar to each other.

Together, these results indicate that RP was the most efficient RNA extraction kit. Moreover, for RP combined with its cognate lysis buffer (RPL), the RNA yield was twice as high as for all other RNA extraction procedures (Tables 2 and 3).

### Comparison of RNA yield determined by RT-qPCR versus 2100 Bioanalyzer

When the extracted RNA was used as a template to amplify *ACTB*, a clear correlation between the Cq values and RNA concentration was observed, with the exception of QZL-EM Run 2 and QZL-RP Run 1, which resulted in very high Cq values (indicative of low amounts of target molecules), compared to moderate RNA concentrations (Table 3). These results are most likely due to a technical error during RT-qPCR, because the Cq values are in correspondence with RNA concentration for Run 2. RE combined with QZL and RLT gave the lowest Cq values (~27), indicating the highest concentrations of target RNA. These results were in agreement with the high RNA concentration, as determined by the 2100 Bioanalyzer. In contrast, RNLs samples resulted in the highest Cq values (> 40), indicating the absence of *ACTB* mRNA from the RNLs, with the exception of the samples extracted with EM (Cq: 27–30), indicating substantial loss of RNA.

### Assessment of the importance of storage medium versus RNA extraction kit for obtaining high RNA yield

After it had been determined that the highest RNA yield was obtained by the procedure that combined storage/lysis in RPL with the RP RNA extraction kit, we questioned which part of the procedure was most important to obtain high RNA yield. Because all three EM-based RNA extraction procedures yielded least RNA, we addressed this question by comparing the RNA yield obtained with the following four combinations: EML-EM, EML-RP, RPL-EM and RPL-RP.

The highest RNA yield was always obtained using the RP RNA extraction kit, irrespective of the storage medium (Table 4), *i*.*e*., ~2000 ng RNA with EML and ~2250 ng with its cognate lysis buffer RPL, compared to the EM RNA extraction kit, *i*.*e*., less than 300 ng with RPL and almost 900 ng with its cognate lysis buffer EML.

**Table 4:** RNA yield for the combinations of storage in EML and RPL and RNA extraction with EM and RP RNA extraction kits.

PMBC storage medium	RNA extraction kit	Yield (ng)
RPL	RP	2257.6 ± 87.2^a^
EML	RP	2004.3 ± 102.3^a^
EML	EM	895.2 ± 163.8^b^
RPL	EM	283.8 ±27.3^c^

Results are means and standards deviation from three independent extractions, in ng. Statistically significant differences among groups are marked with a, b and c (*p* < 0.05) (*t* test for paired samples).

## Discussion

Reliable quantification of gene expression is greatly affected by the quality and quantity of the extracted RNA. Therefore, the way samples are stored preceding RNA extraction as well as the kit of choice to extract RNA are crucial. Thus far, only a limited number of studies compared storage and extraction of RNA from human samples in a methodological manner [19,23,24]. In this study, different storage media (RNL, QZL or cognate lysis buffer (respectively EML, RTL or RPL) and RNA extraction kits (EM, RE or RP) for PBMCs were compared and we determined which conditions provide the highest RNA purity (by means of NanoDrop), integrity (by means of 2100 Bioanalyzer) and quantity (by means of NanoDrop, 2100 Bioanalyzer and RT-qPCR).

RNA integrity and purity must be considered in order to obtain reliable quantification of gene expression [25]. RNA integrity can be measured by using the RNA integrity number (RIN) or the RNA quality number (RQN), both of which indicate for the degree of RNA degradation and which can be determined with a 2100 Bioanalyzer (Agilent Technologies, Santa Clara, CA), a microfluidics-based platform, and a Fragment Analyzer (Applied Biosystems, Foster City, CA), based on capillary electrophoresis, respectively. Both approaches have been shown to be equivalent [26]. Values above 7 for RIN or for RQN are considered to represent high quality and non-degraded RNA.

RNA purity is commonly evaluated by measuring the ratio between the absorbance at 260 nm and 280 nm (A260/A280) absorbance, whereby a value of ~2.0 is generally accepted as indicating that the RNA is free of proteins.

Our study shows that high-quality RNA can be obtained with RE and RP RNA extraction kits regardless of the sample storage medium. All EM extractions also resulted in pure RNA, but with low RIN values (RIN 1). Since RNA analysis was performed with the RNA 6000 Nano kit (Agilent), it may be possible that these results were due to the low concentration of RNA in EM extracts and not due to degradation. For this reason, the analysis was repeated using the RNA 6000 Pico kit (Agilent). Some improvement of the quality of the samples was observed, but RNA was still degraded, with RIN values of 6.6 after QZL storage and less than 2.5 with RNL and EML (S2 File).

In agreement with our results, RE has been reported to provide high-quality RNA from T lymphocytes (RIN value of 10) [27], from bacterial cells (RIN values between 8.75–9.65) [28,29,30], as well as from yeast cells (RIN value of 10) [31]. Lower RIN values of 8 and 7 were obtained for RE-based RNA extraction from human parotid tissue after snap-freezing storage with and without pre-treatment in RNAlater, respectively [32].

Several studies do not consider RNA integrity, and instead assess RNA quality indirectly, through microarray experiments [33], RT-qPCR [34,35,36], or gel electrophoresis and ethidium bromide staining [37,38] or A_260_/A_280_ ratios [39].

The limited number of studies that addressed EM and RP based RNA extraction determined quality of RNA from viruses through RT-qPCR [40,41], and from human bone marrow through A_260_/A_280_ ratios [42].

Both NanoDrop and the 2100 Bioanalyzer correlated well with regard to the relative values obtained for the different procedures that were compared. However, in most cases, NanoDrop indicated RNA concentrations that were 1.1- to 2.5-fold higher than those obtained with the 2100 Bioanalyzer. This is in agreement with the results of [43], reporting RNA concentrations that were 2.3-fold higher according to NanoDrop compared to the 2100 Bioanalyzer results. This may be because NanoDrop also measures separate nucleotides. Indeed, when determining the nucleic acids content of a 2 mM equimolar mixture of dNTPs, the 2100 Bioanalyzer result was negative but Nanodrop measured 666 ng nucleic acids/μl (*i*.*e*., 0.34 mM), for an A_260_/A_280_ value of 1.8.

In addition, the very similar RNA yields, obtained after separate runs of RNA extraction after one day and after 83 days of storage of PBMCs, lends strong support for the reproducibility of our results.

After having shown that purity and integrity of RNA extracts was excellent, with the exception of RNA integrity after EM RNA extraction, and that both runs yielded comparable amounts of RNA, we determined which of three storage conditions yielded most RNA for each of three RNA extraction kits. Together, the results suggest that the optimal storage medium also depends on the subsequent RNA extraction kit. In particular, samples stored in RNAlater resulted in the least amount of RNA extracted. Indeed, according to Qiagen guidelines, RNAlater is not recommended for storage of animal cells–although this is not mentioned in the guidelines of Thermo Fisher Scientific, for the brand of RNAlater that we used. In addition, we stored samples in RNAlater at 4 °C for 83 days, whereas -80 °C is recommended for storage during prolonged time. Despite of prolonged storage at 4 °C, a considerable amount of RNA was still obtained.

Since, according to the manufacturer´s instructions, the RNL supernatant is to be discarded, we assessed possible loss of RNA in the RNLs, which can result from a) prolonged storage of the cells in RNL (at 4 °C), b) from lysis of cells during the centrifugation step needed to remove the RNLs, or c) from incomplete precipitation of cells during centrifugation. For this purpose, RNA was extracted from the RNLs without prior cell lysis.

Quantification of total RNA in the RNLs by means of the 2100 Bioanalyzer indicated 216.7 ng for RE and 274.1 for RP, whereas RT-qPCR for mRNA of the *ACTB* was negative (Cq > 40). The observed difference between both approaches might be due to the presence of extracellular and/or noncoding RNA, not resulting from cell lysis.

However, for EM, we observed higher quantities of RNA in the RNLs (410.7 ng) and moreover, a positive *ACTB* signal (Cq ~ 28.5). This could be due to lysis of cells still present in the RNLs, because in the case of EM, 1 ml of cognate lysis buffer is added, in accordance with the manufacturers’ instructions.

This would indicate that some cells are not precipitated during centrifugation of RNL and that cellular RNA is lost as well, not due to lysis of cells during storage, but due to incomplete recuperation of cells into the RNLp during centrifugation. Indeed, microscopy indicated that many cells were still present in the RNLs (S2 Table).

When comparing the RNA yield for all nine RNA extraction procedures, *i*.*e*., storage in RNL, QZL or cognate lysis buffer (resp. EML, RTL or RPL) combined with the EM, RE or RP RNA extraction kits, the RPL-RP extraction procedure was found to yield twice as much RNA as all other procedures.

In our previous study [31], using storage of *Candida* cells in RNL, the RiboPure RNA Purification Kit also outperformed RE and EM RNA extraction kits, with RP, RE and EM yielding 5.8, 2.6 and 2.2 μg of RNA respectively. Other RNA extraction kits have been shown to provide higher RNA yield. Recently, [16] compared three kits for RNA extraction from human blood samples: the Tempus Spin RNA Isolation Kit (Thermo Fisher Scientific), Norgen Preserved Blood RNA purification Kit I (Norgen Biotek Corp, Ontario, Canada) and MagMax for stabilized blood tubes RNA isolation kit (Thermo Fisher Scientific) and obtained between 8.34 to 12.04 μg of RNA. [44] obtained only 624 ng RNA from 200–300 μl of human blood with the TRI reagent kit (Thermo Fisher Scientific), and the yield of the other two kits tested was still lower. However, because neither study indicated the number of cells that were used for RNA extraction, direct comparison is not possible.[45] extracted ~ 1 μg total RNA from 10^6^ PBMCs, using the miRNeasy Mini Kit (Qiagen), the Total RNA Purification Kit (Norgen Biotek Corp) and the NucleoSpin miRNAs Kit (Macherey-Nagel). This result is in agreement with our study in which we obtained between 200 and 1800 ng RNA using different RNA extraction kits and storage conditions. We obtained up to 800 ng with the RNeasy Mini Kit which is a bit lower than the miRNeasy Mini Kit they used, which might be explained by the fact that the RNeasy Mini Kit selectively excludes all small RNAs (< 200 nt).

To assess which part of the procedure was most important to improve the RNA yield, *i*.*e*. the storage/lysis medium or the RNA extraction kit, we compared the RNA yields obtained with the four possible procedures composed of storage in EML versus RPL buffer combined with EM versus RP RNA extraction kit. The results indicated that the RNA extraction kit has the greatest influence on RNA yield, because whenever RP was used, the highest yield was obtained. Storage in the lysis buffer (EML vs. RPL) also contributed, because storage in the cognate RPL buffer further increased the yield (borderline significantly) from 2004 ng (EML) to 2258 ng (RPL).

In conclusion, when samples can be frozen immediately, this is preferable over storage in RNL at 4 °C. However, a shortcoming of this study is that we failed to assess the performance of RNL storage at -80 °C, as recommended.

In addition, frozen samples (of mammalian cells) are preferably stored in the cognate lysis buffer of the RNA extraction kit. On the other hand, we recently reported that using RNL for the storage of yeast cells gave excellent results [31], which might be explained because of a better precipitation of yeast cells during centrifugation of RNL, compared to mammalian cells.

In conclusion, in this study, RPL-RP offered high-quality RNA and the highest yield of RNA and was the most effective procedure to extract RNA from stored PBMCs. Our results are supported not only by the reproducibility of the experiments in which two different runs of extractions were carried out, but also by RT-qPCR results.

## Supporting information

S1 Table Set up of the study. All kits were carried out in triplicate for each of two extraction runs, *i*.*e*. 6 aliquots were stored per each of 12 combination procedures. PBMC Storage medium: EML: easyMAG lysis buffer; QZL: QIAzol; RLT: RNeasy lysis buffer; RNL: RNAlater pellet; RNLs: RNAlater supernatant; RPL: RiboPure RNA Purification Kit–blood lysis buffer. RNA extraction kits: EM: NucliSENS easyMAG; RE: RNeasy Mini Kit; RP: RiboPure RNA Purification Kit–blood. A: Respectively EML, RLT and RPL for RNA extraction kits EM, RE and RP. B: To add up to a starting volume of 2 ml, as prescribed by the manufacturer. C: No addition of cognate lysis buffer: to check whether RNA could be detected in RNLs supernatant, as a control for possible loss of RNA due to spontaneous lysis of cells during centrifugation of RNL. D: No addition of cognate lysis buffer, because already added prior to storage. E: See Materials & methods, section DNase digestion for detailed description.
(XLSX)

S2 Table Number of PBMCs^a^ in RNAlater pellets and supernatants, after centrifugation at 20,800 *g* for 10 min, counted with a Bürker chamber at a magnification of 400x. a: Starting material consists of 1-ml aliquots PBMCs containing 10^6^ cells. b: Results are means and standard deviations of three independent experiments. c: Pellets were resuspended in 1 ml aliquots of saline before counting.
(DOCX)

S1 Fig Comparison of RNA integrity for three different RNA extraction kits and storage conditions in Run 1, storage of PBMCs during one day. RNA integrity from 10^6^ PBMCs was measured with a 2100 Bioanalyzer and results are shown as A) gel-like image and B) electropherogram profiles. The RIN value is reported on a scale of 1 to 10, whereby values above 7 are considered to represent high quality and non-degraded RNA. vQZL: QIAzol, RNL: RNAlater, RPL: Lysis buffer from RiboPure RNA Purification Kit–blood, EM: NucliSENS easyMAG extraction, RE: RNeasy Mini Kit, RP: RiboPure RNA purification Kit–blood, RLT: Lysis buffer from RNeasy Mini Kit, EML: Lysis buffer from NucliSENS easyMAG extraction.
(TIF)

S2 Fig Comparison of RNA integrity for three different RNA extraction kits and storage conditions in Run 2, storage of PBMCs during 83 days. RNA integrity from 10^6^ PBMCs was measured with a 2100 Bioanalyzer and results are shown as A) gel-like image and B) electropherogram profiles. The RIN value is reported on a scale of 1 to 10, whereby values above 7 are considered to represent high quality and non-degraded RNA. QZL: QIAzol, RNL: RNAlater, RPL: Lysis buffer from RiboPure RNA Purification Kit–blood, EM: NucliSENS easyMAG extraction, RE: RNeasy Mini Kit, RP: RiboPure RNA purification Kit–blood, RLT: Lysis buffer from RNeasy Mini Kit, EML: Lysis buffer from NucliSENS easyMAG extraction.
(TIF)

S1 File Raw data of main figures and tables used in this study. (XLSX)

S2 File RIN values of EM samples obtained with the RNA 6000 Pico kit (Agilent). (XLSX)
