# Supplementary material for: Comparison of procedures for RNA-extraction from peripheral blood mononuclear cells
Source: PLoS One. 2020 Feb 21;15(2):e0229423. doi: 10.1371/journal.pone.0229423 (PMC7034890; doi:10.1371/journal.pone.0229423)
Supplement: S2 Table — a: Starting material consists of 1-ml aliquots PBMCs containing 106 cells. b: Results are means and standard deviations of three independent experiments. c: Pellets were resuspended in 1 ml aliquots of saline before counting. (DOCX) [file pone.0229423.s002.docx]

|  | **PBMCs/ml**^b^ (x 10^5^) |
| --- | --- |
| Pellet^c^ | 5.07 ± 1.23 |
| Supernatant | 3.55 ± 1.13 |
