# Supplementary figures and images for: Comparison of procedures for RNA-extraction from peripheral blood mononuclear cells
Source: PLoS One. 2020 Feb 21;15(2):e0229423. doi: 10.1371/journal.pone.0229423 (PMC7034890; doi:10.1371/journal.pone.0229423)

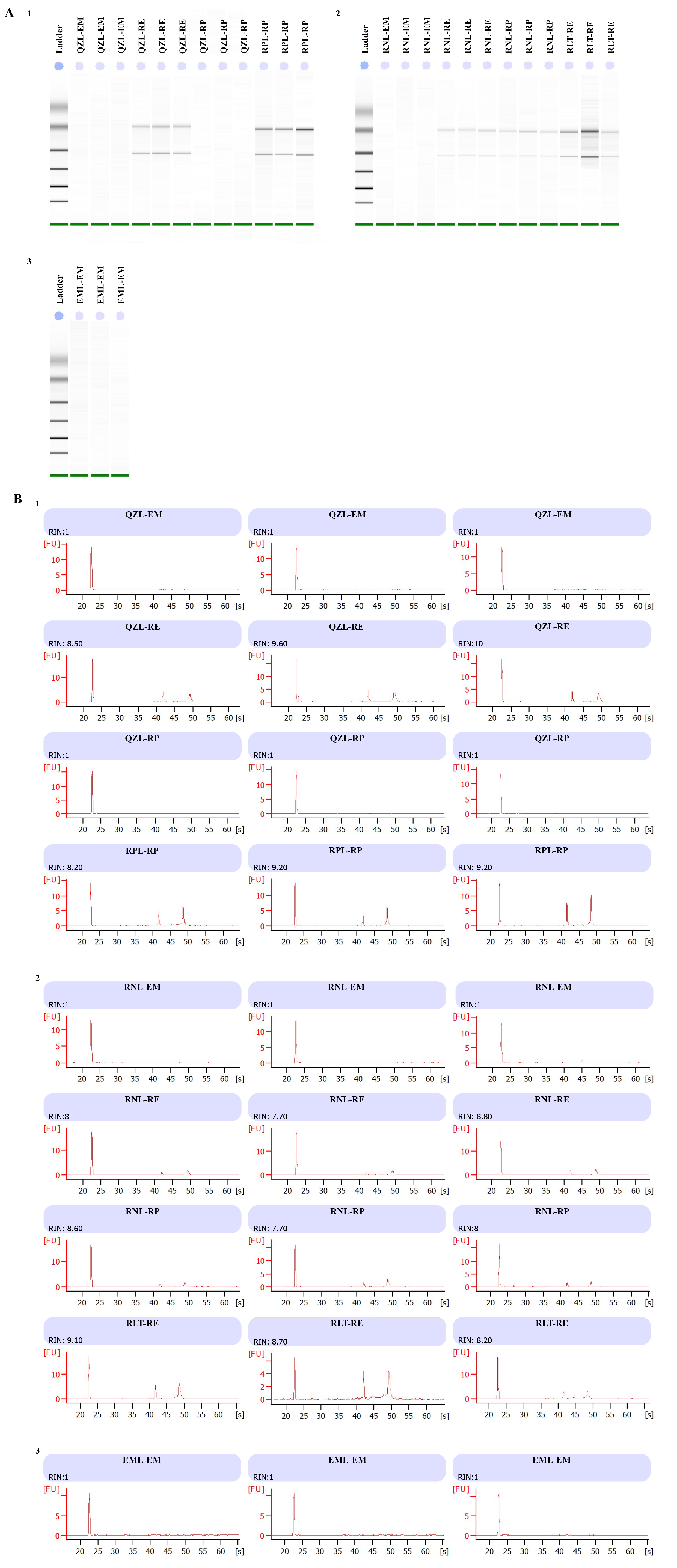

Supplement: S1 Fig — RNA integrity from 106 PBMCs was measured with a 2100 Bioanalyzer and results are shown as A) gel-like image and B) electropherogram profiles. The RIN value is reported on a scale of 1 to 10, whereby values above 7 are considered to represent high quality and non-degraded RNA. vQZL: QIAzol, RNL: RNAlater, RPL: Lysis buffer from RiboPure RNA Purification Kit–blood, EM: NucliSENS easyMAG extraction, RE: RNeasy Mini Kit, RP: RiboPure RNA purification Kit–blood, RLT: Lysis buffer from RNeasy Mini Kit, EML: Lysis buffer from NucliSENS easyMAG extraction. (TIF) [file pone.0229423.s003.tif]

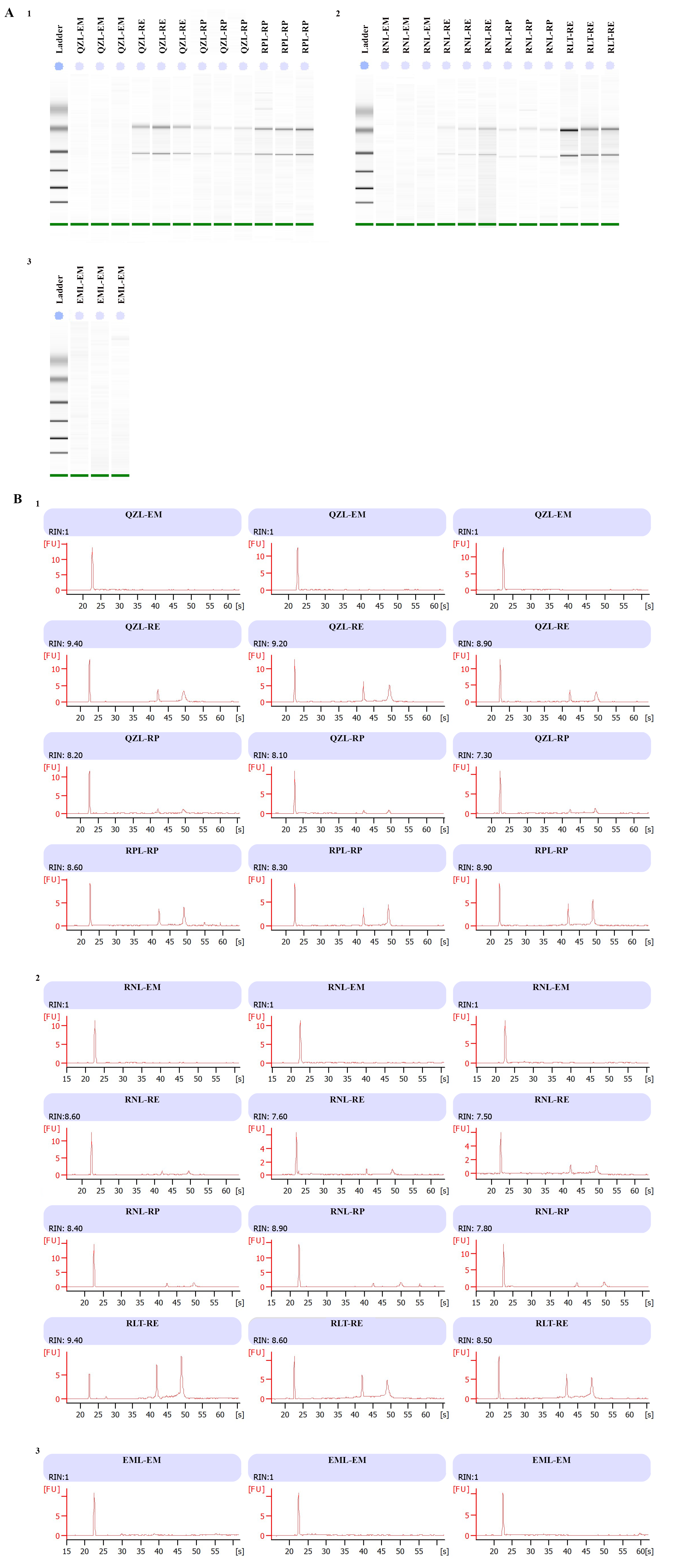

Supplement: S2 Fig — RNA integrity from 106 PBMCs was measured with a 2100 Bioanalyzer and results are shown as A) gel-like image and B) electropherogram profiles. The RIN value is reported on a scale of 1 to 10, whereby values above 7 are considered to represent high quality and non-degraded RNA. QZL: QIAzol, RNL: RNAlater, RPL: Lysis buffer from RiboPure RNA Purification Kit–blood, EM: NucliSENS easyMAG extraction, RE: RNeasy Mini Kit, RP: RiboPure RNA purification Kit–blood, RLT: Lysis buffer from RNeasy Mini Kit, EML: Lysis buffer from NucliSENS easyMAG extraction. (TIF) [file pone.0229423.s004.tif]
